# Drebrin Is Involved in the Life Cycle of Pseudorabies Virus by Regulating the Actin Cytoskeleton

**DOI:** 10.3390/microorganisms13091969

**Published:** 2025-08-22

**Authors:** Kun Xu, Xiao-Han Wang, Yan-Pei Ku, Jie-Yuan Guo, Shu-Han Fan, Miao-Miao Xue, Jiang Wang, Shuang Guo, Jia-Jia Pan, Bei-Bei Chu

**Affiliations:** 1 College of Veterinary Medicine, Henan Agricultural University, Zhengzhou 450046, China; 2 Key Laboratory of Animal Biochemistry and Nutrition, Ministry of Agriculture and Rural Affairs of the People’s Republic of China, Zhengzhou 450046, China; 3 Key Laboratory of Veterinary Biotechnology of Henan Province, Zhengzhou 450046, China; 4 Ministry of Education Key Laboratory for Animal Pathogens and Biosafety, Zhengzhou 450046, China

**Keywords:** Drebrin, actin cytoskeleton, viral life cycle, pseudorabies virus

## Abstract

Pseudorabies virus (PRV), a highly pathogenic alphaherpesvirus, poses a potential threat to public health and safety due to its broad host range and risk of cross-species transmission. Viruses have evolved multiple strategies to exploit host factors for entry into and survival in host cells. Drebrin is an actin-binding protein that restricts rotavirus entry by inhibiting dynamin-mediated endocytosis. However, its role and mechanism in DNA virus infection, particularly in herpesviruses, remain unexplored. In this study, we investigated the role of Drebrin in PRV infection using pharmacological inhibition (BTP−2) and CRISPR-Cas9-mediated gene knockout. Both the Drebrin inhibitor BTP−2 and gene knockout significantly suppressed PRV replication. Intriguingly, Drebrin exhibited stage-specific effects on the viral life cycle: its inhibition enhanced viral internalization during early infection but impaired viral replication at later stages, suggesting that Drebrin plays a complex role in the regulation of PRV infection. PRV infection partially disrupted actin stress fibers and caused an increase in cell size. *Drebrin* knockout also altered the host-cell morphology, reduced the cell surface area, and induced actin cytoskeleton rearrangement, which was further modulated in PRV-infected cells. In summary, our data demonstrate that Drebrin functions as a critical host factor governing the entire PRV life cycle by regulating actin cytoskeleton reorganization.

## 1. Introduction

Pseudorabies is an acute infectious disease caused by the pseudorabies virus (PRV) and leads to significant economic losses in the swine industry [1]. PRV not only infects pigs but also causes acute infection in most mammals [2]. In recent years, variant strains of PRV have been detected in humans and have caused central nervous system diseases, endophthalmitis, and encephalitis [3,4]. These reports suggest that PRV may represent a potential zoonotic pathogen [5]. PRV is an enveloped linear double-stranded DNA virus belonging to the family *Herpesviridae*, genus *Suid herpesvirus*. PRV has been used as a model for studying the molecular biology of herpesviruses and the pathogenesis of herpesvirus-related diseases.

As obligate intracellular pathogens, viruses depend on host cellular machinery for survival. Among various systems within host cells, the cytoskeleton is one of the most essential for the viral life cycle, as it not only facilitates viral invasion and spread to neighboring cells but also aids viruses in evading the host immune system. The host cytoskeleton comprises microfilaments (also called filamentous actin, F-actin), microtubules, and intermediate filaments, among which actin is one of the most abundant cytoskeletal proteins in eukaryotes. The polymerization and depolymerization of actin filaments, as well as the formation of higher-order networks that perform related functions, are regulated by multiple actin-binding proteins (ABPs) [6]. Studies on virus–host interactions have revealed that viruses exploit the host cytoskeletal system, particularly microfilaments and microtubules, to promote their proliferation and survival within host cells [7,8]. Numerous viruses, such as herpes simplex virus-1 (HSV-1), rotavirus (RV), Ebola virus, and respiratory syncytial virus (RSV), utilize actin and ABPs to invade host cells and complete their replication cycles [9,10,11,12].

As one ABP, Drebrin (developmentally regulated brain protein) contains five structural domains: an N-terminal actin depolymerizing factor homology (ADF-H) domain, a coiled-coil domain, a helical domain, a proline-rich region, and a C-terminal domain [13]. Drebrin was initially found to be predominantly expressed in brain tissue, where it participates in neuronal morphogenesis and function, particularly in synapse formation and synaptic plasticity by modulating actin filament reorganization [14,15,16]. Growing evidence has shown that Drebrin is also expressed in other tissues and is involved in diverse biological processes, such as spermatogenesis and tumorigenesis [17,18,19,20]. In addition, studies have revealed that Drebrin regulates the dynamics of the actin cytoskeleton by binding to actin, thereby influencing RNA viral infection. For example, Drebrin inhibited RV entry into host cells by interacting with the viral VP4 protein [21]. Additionally, during HIV-1 (human immunodeficiency virus) infection, Drebrin was recruited to viral envelope glycoproteins and modulated viral infection by regulating the activity of profilin [22]. However, the role of Drebrin in the replication process of DNA viruses, especially herpesviruses, has not been documented. In this study, we employed pharmacological intervention and gene knockout strategies to investigate the role of Drebrin in PRV infection.

## 2. Materials and Methods

### 2.1. Cells and Viruses and Chemical Reagents

Porcine Kidney-15 cells (PK−15), Vero cells, a recombinant strain of PRV−GFP, a virulent PRV isolate strain (PRV-QXX), and antiserum against PRV glycoprotein gB generated by immunizing mice with purified recombinant gB were used as previously described [23]. Anti-GAPDH antibody (60004−1−Ig) and anti-Drebrin antibody (67589-1-Ig) were purchased from Proteintech (Chicago, IL, USA). Lipofectamine 3000 (L3000015), goat anti-mouse IgG (#31431), goat anti-rabbit IgG (#3246), and Alexa Fluor 488-conjugated goat anti-mouse IgG (A-11001) were obtained from Invitrogen (Carlsbad, CA, USA). A Cell Counting Kit−8 (CCK−8, #40203ES76) was purchased from Yeasen BioTechnologies (Shanghai, China). BTP−2 (HY-100831) and Puromycin (P8230) were obtained from MedChemExpress (South Brunswick Township, NJ, USA) and Solarbio (Beijing, China), respectively.

### 2.2. Quantitative Real-Time PCR (RT-qPCR)

Total RNA was extracted using Trizol Reagent (D9108B, TaKaRa, Kusatsu, Shiga, Japan) following the manufacturer’s protocol. First-strand DNA was synthesized using the PrimeScript™ RT Reagent Kit (TaKaRa, RR047A). Gene levels were quantified by SYBR Green-based real-time PCR using SYBR Premix Ex Taq (RR420A, TaKaRa) on a QuantStudio system (QuantStudio5, Thermo Fisher Scientific, Waltham, MA, USA). All reactions were performed in triplicate, with *GAPDH* serving as the reference gene. Reaction specificity was confirmed by melting curve analysis, which showed single-peak amplification products in all cases. Relative gene expression was calculated by the 2^−ΔΔCt^ method. The primers of *GAPDH*, *PRV-gB*, and *PRV-TK* were used as previously described [23]. The primers used for *Drebrin* were as follows: Sus-Drebrin-Fw, TTGCCCAGCGACCTGATAAC, Sus-Drebrin-Rv, TATGAAAGGGCAGTACGGACG. To determine the PRV viral load, a 187 bp fragment of the glycoprotein H (gH) gene was amplified by PCR and cloned into a pGEM-T vector to generate a standard curve. Serial 10-fold dilutions of this recombinant plasmid were used as the quantification standard. Viral genome copy numbers in test samples were interpolated from the standard curve.

### 2.3. Western Blotting

Whole-cell lysates were extracted with RIPA buffer (P0013B, Beyotime, Shanghai, China) supplemented with protease and phosphatase inhibitors (HY-K0010 and HY-K0022, MedChemExpress). The protein concentrations in the lysates were quantified with a BCA Protein Assay Kit (BCA01, DingGuo Biotech, Beijing, China), detected with a microplate reader (Awareness Technology Inc., Palm City, FL, USA). Protein lysates were separated by SDS-PAGE and transferred to nitrocellulose membranes (Millipore, Burlington, MA, USA). To minimize nonspecific binding, membranes were blocked in 5% non-fat milk (Sangon, Shanghai, China) for 1 h. Primary antibodies (anti-GAPDH and anti-Drebrin) and antiserum against PRV glycoprotein gB were diluted in blocking buffer and incubated with the membranes at 4 °C overnight with gentle agitation. After extensive washing, membranes were probed with horseradish-peroxidase-conjugated secondary antibody for 1 h at room temperature. Subsequently, the protein signals were examined by using Luminata Crescendo Western HRP Substrate (Millipore, WBLUR0500, MA, USA) and captured on a GE AI600 chemiluminescence imaging system (General Electric Company, Boston, MA, USA). Band intensities were quantified using ImageJ software (ImageJ 1.43u/Java 1.6.0_10 (32-bit)).

### 2.4. Immunofluorescence Assay (IFA)

Differently treated PK−15 cells cultured on glass coverslips were fixed with 4% paraformaldehyde for 10 min. After washing with phosphate-buffered saline (PBS), the cells were permeabilized with PBS containing 0.1% Triton X-100 for 10 min and then blocking with bovine serum albumin (BSA) in PBS for 60 min. For localization of Drebrin, cells were incubated with primary mouse anti-Drebrin mAb at 4 °C overnight. After washing with PBS, the cells were further incubated with fluorescent secondary antibodies (Alexa-Fluor-488-conjugated goat anti-mouse A-11001, Invitrogen, CA, USA) for 1 h. For the detection of PRV-gB, PRV-QXX-infected PK−15 cells were incubated with antiserum against PRV glycoprotein gB and then Alexa-Fluor-488-conjugated goat anti-mouse. For the detection of actin morphology, the cells were directly labeled with Rhodamine-phalloidin (R415, Invitrogen, CA, USA). The cells were finally washed in PBS and mounted in ProLong Diamond with DAPI (#P36971, Invitrogen). Then, the slides were examined under a Zeiss LSM 800 confocal microscope (Cral Zeiss, Oberkochen, Germany). The relative fluorescence intensity was measured by ImageJ software.

### 2.5. Generation of Drebrin Gene Knockout Cell Lines Using CRISPR-Cas9

To establish stable *Drebrin*-deficient cell lines, a lentivirus-mediated CRISPR/Cas9 gene silencing system was employed as previously described [24]. The target sequence (5′-CACCGTTCGGTCCGGCCCGCAGCA-3′) was designed against the *Drebrin* coding region and cloned into the lentiCRISPR v2 vector (Addgene, #52961, Watertown, MA, USA) according to the manufacturer’s instructions. This construct was co-transfected with packaging plasmids pMD2.G (Addgene, #12259) and psPAX (Addgene, #12260) into HEK293T cells. Lentiviral supernatants were harvested 48 h post-transfection and used to infect cells in the presence of puromycin (4 μg/mL) for seven days. Single-cell clones were isolated by serial dilution in 96-well plates and verified by sequencing (Sangon Biotech Co., Ltd., Shanghai, China) and immunoblot analysis.

### 2.6. Flow Cytometry (FCM)

To assess the replication of PRV, target cells were infected with PRV-GFP with indicated MOIs (multiplicities of infection). Then, cell monolayers were treated with 0.25% trypsin-EDTA (25200-072, GIBCO, Grand Island, NY, USA) to dislodge the cells from the flask surface. Single-cell suspensions were prepared in PBS for flow cytometry analysis. Infection of cells was determined by counting the number of GFP-positive cells on CytoFLEX (Beckman, Atlanta, GA, USA). A minimum of 10,000 events were acquired per sample, with uninfected cells serving as a negative control for gating purposes. Data processing and statistical analysis were performed using CytExpert software 2.0, with results expressed as the percentage of GFP-positive cells relative to total live cells.

### 2.7. Virus Titer Detection

Virus titers were evaluated using the 50% tissue culture infective dose (TCID_50_) assay in Vero cells. Cells were seeded in 96-well plates at a density of 1 × 10^4^ cells per well. On the next day, ten-fold serial dilutions of viruses collected from different treatments were inoculated onto the cell monolayers in octuplicate wells for 1 h at 37 °C. The excess viral inoculum was removed and 200 μL of maintenance medium (2% FBS/DMEM) was added; then, the cells were further incubated at 37 °C for 3–7 days. The cells were then examined daily for characteristic cytopathic effects (CPEs) under microscopy, and the TCID_50_ values were calculated according to the Reed–Muench method [25].

### 2.8. Virus Binding and Entry Assays

For viral attachment assays, PK−15 cells were co-incubated with BTP−2 (600 nM) and PRV-QXX (MOI = 10) at 4 °C for 2 h to simulate virus adsorption under low-temperature conditions. Unbound viral particles were removed by washing cells, and then viral genomic DNA was extracted and the copy number of the PRV genome was evaluated by qRT-PCR analysis.

For viral entry assays, PK−15 cells were firstly incubated with PRV-QXX (MOI = 10) at 4 °C for 1 h to allow virus adsorption. Subsequently, the unbound virus was removed, and medium containing 600 nM BTP−2 was added. Cells were incubated at 37 °C for another hour to allow viral entry; then, cells were washed to remove the residual virions on the plasma membrane, and viral entry was detected by qRT-PCR analysis of viral genome copy numbers. In addition, the sgDBN and sgDBN cells were incubated with PRV-QXX (MOI = 10) at 4 °C for 1 h to allow virus adsorption. The unbound virus was removed, cells were incubated at 37 °C for another 1 h to allow viral entry, and then cells were washed to remove the residual virions on the plasma membrane. Finally, viral entry was evaluated by qRT-PCR and TCID_50_.

### 2.9. Statistical Analysis

For statistical validation, each experiment was repeated independently at least three times. Data are reported as arithmetic means with standard error of the mean (SEM) as the measure of dispersion. We performed parametric analyses by unpaired two-tailed *t*-test (for testing of two samples) or one-way ANOVA (for testing of more than two samples) using GraphPad Prism 8.0.2. Significance levels relative to the corresponding control were denoted as non-significant (ns) for *p* > 0.05, * *p* < 0.05, ** *p* < 0.01, and *** *p* < 0.001.

## 3. Results

### 3.1. Changes in the Drebrin Expression Levels During PRV Infection in PK−15 Cells

**Figure 1:**
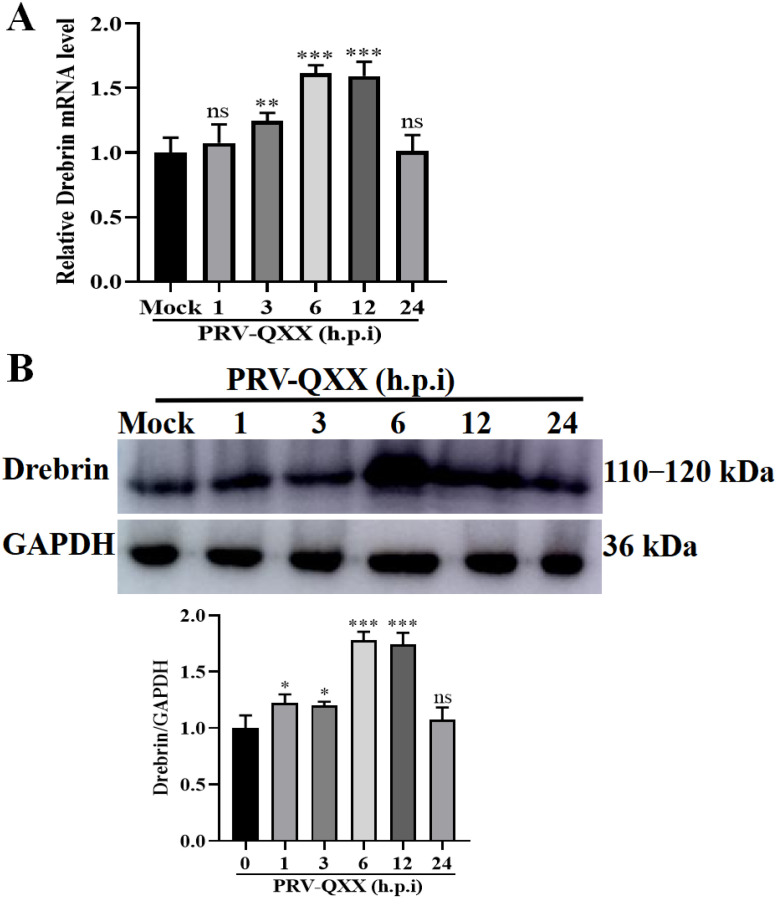
Expression of Drebrin response to PRV infection. PK−15 cells were infected with PRV-QXX (MOI = 1) for indicated times. *Drebrin* mRNA and protein levels were evaluated via qRT-PCR (**A**) and Western blot (**B**), respectively. GAPDH was used as the loading control. Gray value analysis using ImageJ. Significance levels relative to the corresponding control were denoted as non-significant (ns) for *p* > 0.05, * *p* < 0.05, ** *p* < 0.01, and *** *p* < 0.001.

To explore the role of Drebrin during PRV infection, we analyzed the expression of Drebrin response to PRV infection in PK−15 cells. Cultured cells were infected with the PRV-QXX strain and incubated for various durations, ranging from 0 to 24 h; subsequently, total RNA and protein were extracted to detect the Drebrin mRNA and protein levels. As shown in Figure 1A, the level of *Drebrin* mRNA increased by 1.2-fold at 3 h and reached 1.7-fold at 6–12 h.p.i (hours post infection), which was significantly different from that in mock-infected cells. Then, mRNA levels of *Drebrin* reverted and were identical to the mock group at 24 h. Accordingly, a markedly elevated Drebrin protein level was also observed at 6–12 h.p.i (Figure 1B). This increase in Drebrin at both the mRNA and protein level suggested that PRV infection upregulated Drebrin expression.

### 3.2. Inhibition of Drebrin Reduced PRV Infection

**Figure 2:**
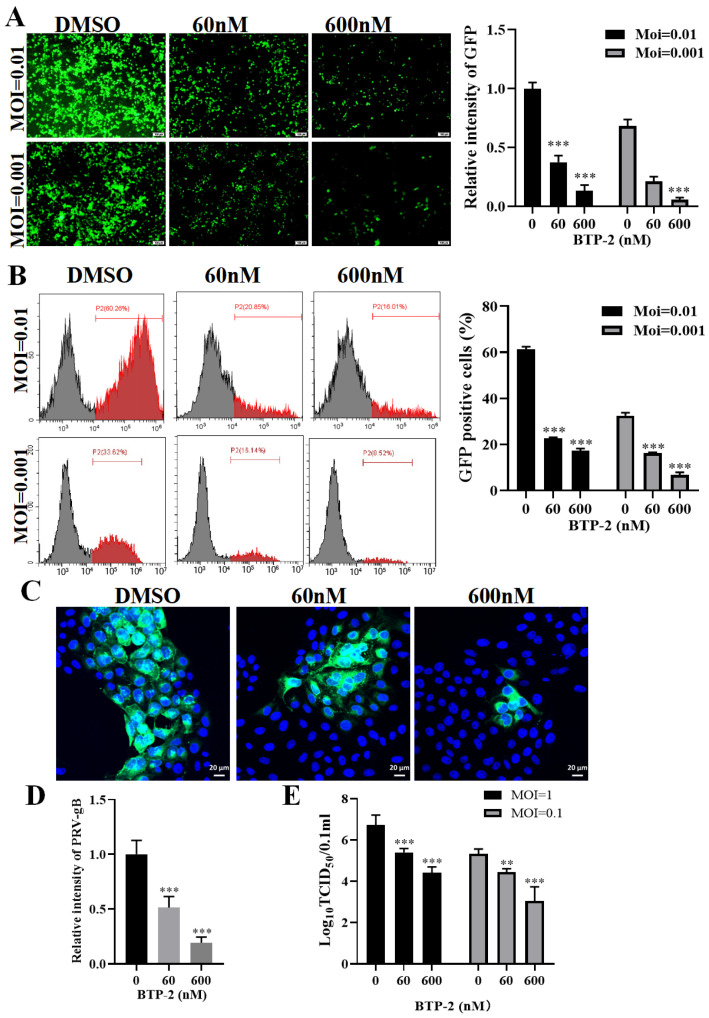
Inhibition of Drebrin by BTP−2 restricted PRV infection. (**A**) PK−5 cells were incubated with the indicated concentration of BTP−2 for 4 h and infected with PRV-GFP (MOI = 0.01 and 0.001) for 24 h with the continuous presence of BTP−2; then, cells were observed under a fluorescence microscope, and the fluorescence intensity of GFP was measured by ImageJ. Scale bar, 100 μm. (**B**) PK−15 cells were treated as in (**A**), and the GFP-positive cells were measured by flow cytometry. (**C**) PK−15 cells were treated as in (**A**) and infected with PRV-QXX (MOI = 0.1) for 24 h; then, cells were fixed with 4% PFA and stained with anti-gB antibody for viral glycoprotein gB (green) and DAPI for nucleus (blue). The images were captured by microscope (Zeiss LSM 800). Scale bar, 20 μm. (**D**) The fluorescent intensity of PRV-gB in (**C**) was measured by ImageJ. (**E**) Cells were treated as in (**A**) and infected with PRV-QXX (MOI = 0.1 and 1) for 24 h; then, the viral titer was determined by TCID_50_ assay. Significance levels relative to the corresponding control were denoted as ** *p* < 0.01, and *** *p* < 0.001.

To assess the roles of Drebrin in PRV proliferation, we firstly explored the effect of the Drebrin inhibitor BTP−2 on PRV proliferation in PK−15 cells. PK−15 cells were first incubated with the indicated concentration of BTP−2 for 4 h, infected with PRV-GFP at different MOIs, and then incubated with the same concentration of BTP−2 for 24 h, followed by fluorescent microscope (Figure 2A) and flow cytometry (Figure 2B) analysis. Compared with control cells, BTP−2-treated cells exhibited significantly reduced PRV-GFP infectivity, as evidenced by both diminished GFP signal intensity (*p* < 0.001) (Figure 2A) and a reduced percentage of GFP-positive cells (*p* < 0.001) (Figure 2B). The inhibitory effect of BTP−2 on PRV-GFP infection gradually strengthened with the decrease in infection dose. To further characterize BTP−2’s antiviral activity, we assessed its impact on PRV-QXX replication using IFA and viral titration assays. IFA revealed a dose-dependent suppression of viral protein gB expression (Figure 2C,D). Viral titration by TCID_50_ assay confirmed this inhibitory effect, demonstrating a progressive decline in progeny virion production with increasing BTP−2 concentrations (Figure 2E). These results showed that BTP−2 inhibited PRV infection in a dose-dependent manner.

### 3.3. Depletion of Drebrin Inhibited PRV Replication in PK−15 Cells

**Figure 3:**
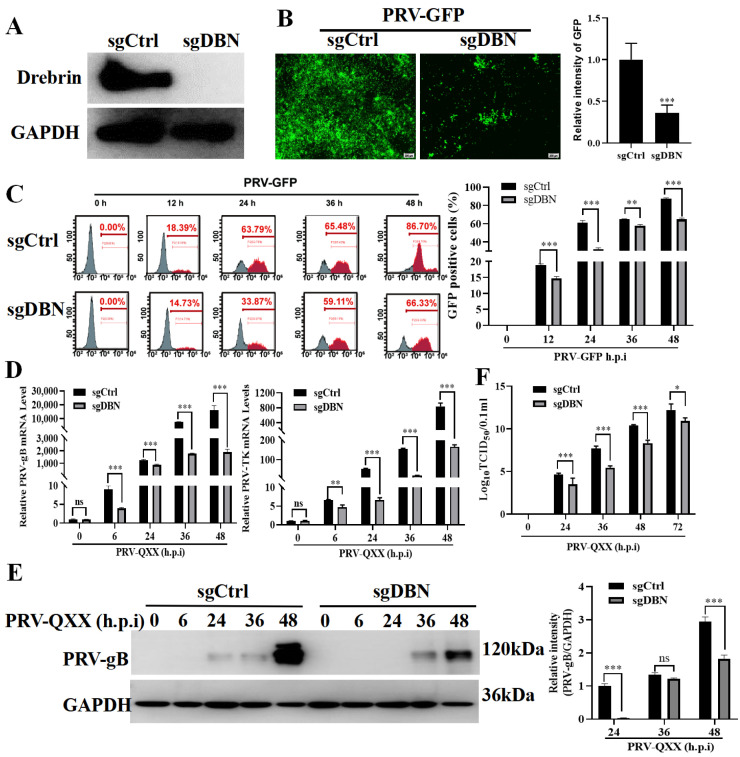
Knockout of *Drebrin* inhibited PRV replication. (**A**) The *Drebrin* knockout cell line was constructed, and the efficiency of knockout was assayed by Western blot. GAPDH was used as the loading control. (**B**) The sgCtrl and sgDBN cells were infected with PRV−GFP (MOI = 0.01) for 24 h; then, the cells were observed under a fluorescence microscope, and the fluorescence intensity of GFP was measured by ImageJ. Scale bar, 200 μm. (**C**) sgCtrl and sgDBN cells were infected with PRV-GFP (MOI = 0.01) for indicated times, and the GFP-positive cells were measured by flow cytometry. (**D**) The sgCtrl and sgDBN cells were infected with PRV-QXX (MOI = 1) for indicated times, and the *PRV-gB* and *PRV-TK* genes level were determined by RT-qPCR. (**E**) The sgCtrl and sgDBN cells were treated as in (**D**), and then the cells were collected and subjected to Western blot for the analysis of PRV-gB levels. GAPDH served as the loading control. (**F**) The sgCtrl and sgDBN cells were infected with PRV-QXX (MOI = 1) for different times, the viruses were harvested with three freeze–thaw cycles, and then the viral titers were analyzed by TCID_50_ assay. Significance levels relative to the corresponding control were denoted as non-significant (ns) for *p* > 0.05, * *p* < 0.05, ** *p* < 0.01, and *** *p* < 0.001.

Considering that the BTP−2 used to inhibit Drebrin in this study can also affect calcium signaling, we ablated *Drebrin* to verify its roles in PRV proliferation. CRISPR-Cas9 technology was used to generate the *Drebrin* knockout PK−15 cell line, and the efficiency of knockout was assessed by Western blot (Figure 3A). Then, we infected PK−15 (sgCtrl) and sgDrebrin (sgDBN) PK−15 cells with PRV-GFP and found that depletion of *Drebrin* decreased PRV-GFP proliferation, as indicated by the fluorescent microscope and flow cytometry analysis of GFP-positive cells (Figure 3B,C). We next infected sgCtrl and sgDBN with PRV-QXX to further analyze the PRV replication by different methods. Firstly, we found that the expression of viral genes *gB* and *TK* were both inhibited in sgDBN cells (Figure 3D). Consistent with the viral gene level, the expression of viral glycoprotein gB was also reduced at 24 h.p.i and 48 h.p.i (Figure 3E). In addition, the viral yield was significantly lower in sg*DBN* cells than in control cells during different times of infection (Figure 3F). These results demonstrated that depletion of *Drebrin* inhibited PRV replication, consistent with the inhibitor-based findings.

### 3.4. Drebrin Is Involved in the Whole Life Cycle of PRV Infection

**Figure 4:**
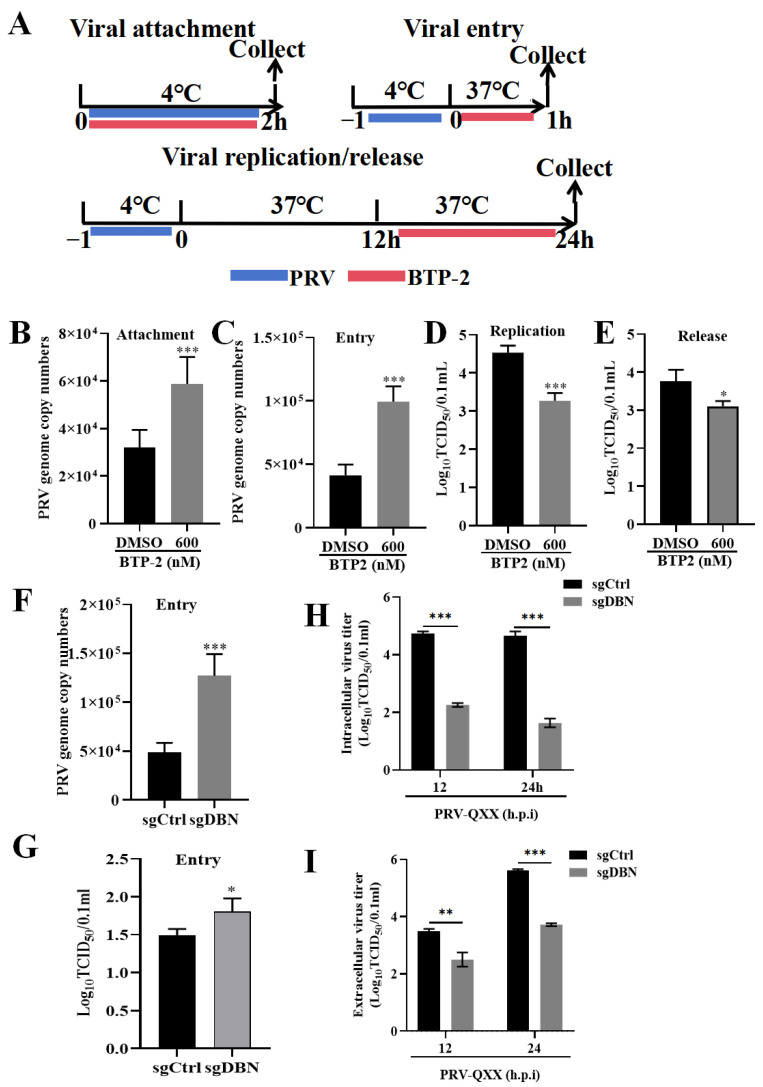
Effects of Drebrin on the different stages of PRV infection. (**A**) Schematic timeline for the time-of-addition assays. (**B**) BTP−2 (600 nM) was co-incubated with PRV-QXX (MOI = 10) at 4 °C for 2 h to simulate virus adsorption under low-temperature conditions in PK−15 cells. Unbound viral particles were removed, viral genomic DNA was extracted, and the copy numbers of the viral genome were evaluated by qRT-PCR. (**C**) PK−15 cells were firstly incubated with PRV-QXX (MOI = 10) at 4 °C for 1 h to allow virus adsorption. Subsequently, unbound virus was removed, and medium containing 600 nM BTP−2 was added. Cells were treated at 37 °C for 1 h, and viral genome copy number was evaluated by qRT-PCR. (**D**) PK−15 cells were incubated with PRV−QXX (MOI = 1) at 4 °C for 1 h, and the inoculum was then removed and replaced with maintenance medium containing 2% FBS/DMEM. Cells were incubated at 37 °C for 12 h to permit viral entry and early replication events. Subsequently, BTP2 was added, and cells were incubated at 37 °C for another 12 h. Then, the virus was collected by being frozen and thawed three times, and the TCID_50_ assay was performed to evaluate the virus titer. (**E**) PK−15 cells were treated as in (**D**): the supernatant was collected and TCID_50_ was performed to evaluate the extracellular viral yield. (**F**) The sgCtrl and sgDBN cells were incubated with PRV-QXX at 4 °C for 2 h, cells were rinsed 3 times with ice-cold PBS and incubated with maintenance solution at 37 °C for 30 min, and then the PRV genome copy number was determined by RT-qPCR. (**G**) Cells were treated as in (**D**): viruses were harvested with three freeze–thaw cycles, and then the viral titers were analyzed by TCID_50_ assay. (**H**,**I**) The sgCtrl and sgDBN cells were incubated with PRV−QXX at 37 °C for 1 h, cells were rinsed three times with PBS, and the medium was replaced with 2% FBS/DMEM to maintain growth for 12 h and 24 h. Then, the cells and supernatant were collected to analyze the intracellular and extracellular viral yield by the TCID_50_ assay, respectively. Significance levels relative to the corresponding control were denoted as non-significant * *p* < 0.05, ** *p* < 0.01, and *** *p* < 0.001.

To deepen our understanding of the impact of Drebrin on the PRV life cycle, we thoroughly dissected the stages in which Drebrin played a pivotal role. On one hand, we analyzed the effects of BTP−2 on different stages of the PRV life cycle by time-of-addition assay. The initial phase of viral infection involves the attachment and entry of the virus into host cells. PK−15 cells were incubated with BTP−2 and PRV-QXX at 4 °C for 2 h, and qPCR analysis revealed that the viral genome copy number was significantly higher in the group treated with BTP2 compared to the control group, suggesting that BTP2 may facilitate the adsorption of the virus at this concentration (Figure 4B). We next carried out a viral entry assay. Quantification of the viral genome copy numbers by RT-qPCR indicated that the BTP−2 treatment enhanced the viral entry compared with control group (Figure 4C). In the replication and assembly stage, the virus titer assay demonstrated that BTP−2 treatment led to a decrease in viral yield compared with the DMSO treatment (Figure 4D). In addition, compared with the control group, the BTP−2 treatment resulted in a reduced viral production in the supernatant, as indicated by the TCID_50_ assay (Figure 4E). These data indicated that Drebrin might function differently in the PVR life cycle.

On the other hand, we used the *Drebrin* knockout cell line to further investigate the comprehensive roles of Drebrin in the PRV life cycle. Entry into host cells is the initial and key stage for viral infection. Therefore, we first tested the effect of *Drebrin* knockout cells on the PRV entry stage, and both qPCR and TCID_50_ assays showed that knockout of *Drebrin* resulted in an increased viral yield (Figure 4F,G). Then, we tested the effect of knockout of the *Drebrin* gene on the viral replication and release. The intracellular and extracellular viruses were collected at 12 h and 24 h of infection, and the viral production was measured by TCID_50_ assay, which showed that the progeny viral titers in *Drebrin* knockout were significantly lower than those in control cells (Figure 4H,I). The inhibitor treatment and gene knockout strategy both showed that inhibition of Drebrin facilitated PRV internalization into host cells at the early stage, whereas it inhibited PRV replication or release at the later stage of the life cycle. In summary, these data implied that Drebrin was involved in the entire life cycle of PRV infection and played distinct functions.

### 3.5. Drebrin Induced the Dynamic Change in Actin Cytoskeleton During PRV Infection

**Figure 5:**
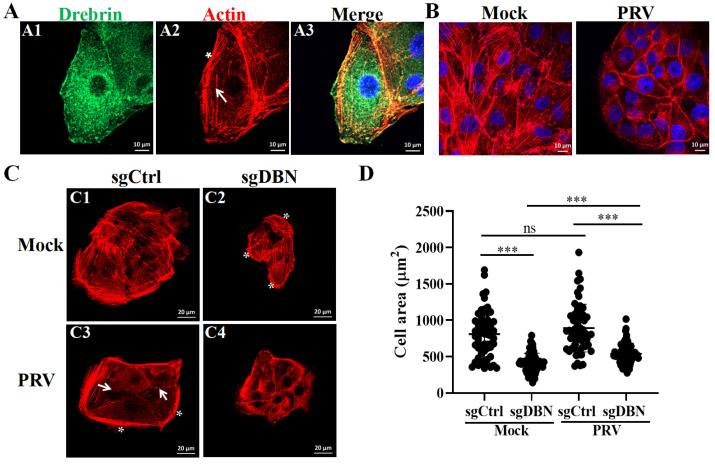
Drebrin remodeled the dynamics of the actin cytoskeleton during PRV infection. (**A**) PK−15 cells were fixed with 4% PFA then subjected to IFA to detect Drebrin (green) and actin (red). Drebrin was stained with primary antibody (Mouse Anti-Drebrin antibody) and second antibody (Alexa-Fluor-488-conjugated goat anti-mouse). The actin was directly stained with rhodamine-phalloidin. The nuclei were stained with DAPI (blue). Scale bar, 10 μm. The white arrowhead indicates the actin stress fibers, and the asterisk presents the actin bundles. (**B**) PK−15 cells were either uninfected or infected with PRV-QXX (MOI = 1) then directly stained with rhodamine-phalloidin for actin (red) and DAPI for the nucleus (blue). Scale bar, 10 μm. (**C**) The sgCtrl and sgDBN cells were infected with PRV-QXX (MOI = 1) and then subjected to IFA to detect actin morphology by directly staining with rhodamine-phalloidin. The arrowhead indicates the actin-based spiky protrusions, and the asterisk presents the actin bundles. Scale bar, 20 μm. These images were all captured by a Zeiss LSM 800 microscope. (**D**) The cell areas of (**C**) were measured by ImageJ. For each group, 60 cells were randomly measured. Significance levels relative to the corresponding control were denoted as non-significant (ns) for *p* > 0.05, and *** *p* < 0.001.

Drebrin is an ABP that binds to F-actin and is involved in the regulation of actin cytoskeletal rearrangement, especially the polymerization process [13]. Hereby, we analyzed the distribution pattern of Drebrin in PK−15 cells. Meanwhile, we stained the actin with Rhodamine-phalloidin to investigate the colocalization of Drebrin and actin. As shown in Figure 5(A1), Drebrin predominantly diffused in the cytoplasm, especially at the periphery of the nucleus and at certain regions in the plasm membrane. The actin cytoskeleton showed distinct structures in PK−15 cells, such as being diffused in the cytoplasm and forming actin stress fibers and actin bundles (Figure 5(A2)). Notably, we found that Drebrin partially colocalized with actin in PK−15 cells, predominantly at the actin stress fiber regions and also a small amount at the periphery of the nucleus (certain yellow regions of Figure 5(A3)).

Not surprisingly, viruses have developed strategies to reorganize the actin cytoskeleton to successfully infect host cells. The dynamics change in actin, and its functions are regulated by multiple actin-binding proteins in virus infection. In a previous study, using the compound library targeting actin and actin-associated proteins, our group showed that the actin cytoskeleton played essential roles in PRV infection [26]. Herein, we found that PRV infection induced the rearrangement of the actin cytoskeleton, the actin stress fibers were disrupted, and actin accumulated more at the periphery of cell membranes (Figure 5B). In addition, we wondered if Drebrin affects the dynamic changes in the actin cytoskeleton during PRV infection. To this aim, sgCtrl and sgDBN cells were infected with PRV-QXX and then subjected to IFA assay for observing the morphology of the actin cytoskeleton. Uninfected control cells were used as the control (mock). As shown in Figure 5(C1), the actin diffused in the cytoplasm and formed stress fibers and dorsal actin bundles (Figure 5(C1)), which was consistent with the localization pattern in PK−15 cells (Figure 5(A2)). Compared with the control cells, PRV infection induced the rearrangement of the actin cytoskeleton, the formation of stress fibers was partially disrupted, actin-based spiky protrusions (arrowheads) were generated at the edge of cells, and there were thick dorsal actin bundles at the outer rim of the cell population (asterisk) (Figure 5(C3)). In addition, we found that the area of cells infected with PRV were bigger than the mock cells, but the difference did not reach statistical significance (Figure 5D).

As expected, depletion of Drebrin also induced a dynamic change in the actin cytoskeleton (Figure 5(C2)), and the formation of stress fibers was partially disrupted as in PRV-infected sgCtrl cells; in addition, actin-based spiky protrusions were formed at the edge of cells. Meanwhile, lamellipodia and actin arcs appeared at the edge extension of some cells, as indicated by the asterisk in Figure 5(C2). Additionally, in the *Drebrin*-depleted cells infected with PRV, the actin cytoskeleton was dramatically remodeled, as indicated by the disappearance of actin stress fibers, and the depolymerized actin proteins diffused in the cytoplasm and accumulated at the membranes (Figure 5(C4)). Strikingly, both in the mock and PRV infected cells, the *Drebrin*-depleted cells had a significantly smaller surface area than the control cells (Figure 5D). However, the area of the sgDBN cells infected with PRV was significant bigger than the mock-sgDBN cells. These markable changes in the actin dynamics and cell surface area were associated with the PRV infection.

## 4. Discussion

Growing evidence indicates that actin interacts with various ABPs to form higher-order structures critical for diverse cellular functions, including viral infection. As a typical ABP, Drebrin participated in virus RNA infection, as the depletion of Drebrin increased the infection of RV and HIV-1 [21,22]. In this study, our data showed that knockout Drebrin inhibited the DNA virus PRV replication, implying that Drebrin had a different effect on virus infection than in previous reports. Nevertheless, by meticulously dissecting, we found a dual role of Drebrin in the PRV life cycle: enhanced early internalization yet restricted late-stage replication, suggesting it plays complicated roles in the PRV life cycle. Interestingly, Drebrin facilitated the entry of RV and HIV-1 into host cells, but it has no effect on the internalization of Zika virus or Dengue virus [21]. In addition, Drebrin restricted RV entry by inhibiting dynamin-mediated endocytosis, whereas Drebrin inhibited HIV-1 entry by stabilizing HIV-1-triggered F-actin polymerization. These complementary data indicate that Drebrin participates in different virus infections with stage-specific roles, possibly via different mechanisms.

Drebrin is an F-actin-binding protein essential for neuronal plasticity because it is able to change the properties of actin filaments and thereby modulates dendritic spine morphology [27]. Our data demonstrated that *Drebrin* ablation altered cell morphology, reduced surface area, and induced actin reorganization—a phenotype consistent with its role in neurite outgrowth and synaptic plasticity [27]. PRV infection further exacerbated these changes, implying viral exploitation of Drebrin-mediated cytoskeletal remodeling. Similar mechanisms have been reported for other herpesviruses. HSV-1 and Marek’s Disease Virus hijacked Rho GTPases to manipulate actin dynamics for viral entry and cell-to-cell spread [28,29]. The observed cytoskeletal disruption in *Drebrin*-knockout cells likely impairs PRV’s ability to establish intracellular trafficking routes or budding sites, ultimately limiting viral yield. Remarkably, Drebrin is known to stabilize the F-actin network, and its knockout may mimic actin-disrupting effects, thereby accelerating PRV internalization. Conversely, the suppression of late-stage replication suggested Drebrin was essential for viral factory formation or virion assembly, possibly by maintaining cytoskeletal integrity for efficient viral genome replication or capsid transport [30]. This dichotomy of functions underscores the context-dependent functions of actin regulators in viral infection.

Viral proteins can bind and interact with host factors to facilitate viral replication. For example, the PRV envelope glycoprotein gB interacts with host cell surface receptors (e.g., heparan sulfate proteoglycans or nectin-1) to mediate viral entry. This binding triggers membrane fusion, allows the virus to enter, and initiates infection. In addition, PRV glycoprotein D interacts with the host factor THBS3 to promote viral attachment, fusion, and entry [31]. Studies have revealed that Drebrin interacts with the capsid protein VP4 of RV, thereby inhibiting viral entry into host cells [21]. Herein, we found that knockout *Drebrin* inhibited the expression of the PRV gB protein, and we will further analyze the interaction between Drebrin and PRV gB or other PRV proteins by molecular docking, gene mutation, and co-immunoprecipitation assays, as well as the effects of their interaction on PRV infection. Furthermore, given Drebrin’s roles in cytoskeletal remodeling, immune regulation, and calcium signaling [32], future work should delineate the precise mechanisms by which it governs PRV pathogenesis.

In summary, our study suggests that Drebrin functions as a critical host factor that spatiotemporally orchestrates PRV infection by regulating the actin cytoskeleton. Notably, pharmacological inhibition or genetic ablation of Drebrin has been shown to enhance RV infection across cell types and animal models [21]. In contrast to these findings, our study demonstrated the stage-specific inhibition effects of Drebrin in PRV infection, suggesting caution in targeting it for antiviral therapy. Additionally, while BTP−2 suppressed PRV replication, its early pro-entry effect could exacerbate initial infection. This mirrors challenges faced with other cytoskeletal-targeting drugs, which might exhibit broad antiviral activity but risks off-target toxicity. Alternatively, selectively modulating Drebrin’s interactions with late-stage viral effectors (e.g., PRV tegument proteins) might offer a safer strategy [33].

## Data Availability

The original contributions presented in this study are included in the article. Further inquiries can be directed to the corresponding authors.
